# The genetic basis of female multiple mating in a polyandrous livebearing fish

**DOI:** 10.1002/ece3.435

**Published:** 2013-01-10

**Authors:** Jonathan P Evans, Clelia Gasparini

**Affiliations:** Centre for Evolutionary Biology, School of Animal Biology (M092), The University of Western AustraliaCrawley, 6009, WA, Australia

**Keywords:** Cryptic female choice, genetic correlation, polyandry, promiscuity

## Abstract

The widespread occurrence of female multiple mating (FMM) demands evolutionary explanation, particularly in the light of the costs of mating. One explanation encapsulated by “good sperm” and “sexy-sperm” (GS-SS) theoretical models is that FMM facilitates sperm competition, thus ensuring paternity by males that pass on genes for elevated sperm competitiveness to their male offspring. While support for this component of GS-SS theory is accumulating, a second but poorly tested assumption of these models is that there should be corresponding heritable genetic variation in FMM – the proposed mechanism of postcopulatory preferences underlying GS-SS models. Here, we conduct quantitative genetic analyses on paternal half-siblings to test this component of GS-SS theory in the guppy (*Poecilia reticulata*), a freshwater fish with some of the highest known rates of FMM in vertebrates. As with most previous quantitative genetic analyses of FMM in other species, our results reveal high levels of phenotypic variation in this trait and a correspondingly low narrow-sense heritability (*h*^2^ = 0.11). Furthermore, although our analysis of additive genetic variance in FMM was not statistically significant (probably owing to limited statistical power), the ensuing estimate of mean-standardized additive genetic variance (*I*_A_ = 0.7) was nevertheless relatively low compared with estimates published for life-history traits across a broad range of taxa. Our results therefore add to a growing body of evidence that FMM is characterized by relatively low additive genetic variation, thus apparently contradicting GS-SS theory. However, we qualify this conclusion by drawing attention to potential deficiencies in most designs (including ours) that have tested for genetic variation in FMM, particularly those that fail to account for intersexual interactions that underlie FMM in many systems.

## Introduction

The selective forces fueling the evolution of female multiple mating (FMM), where females mate with two or more males within a single reproductive episode, remain largely unresolved in many species (Slatyer et al. 2012). Among the various hypotheses proposed to explain FMM, one that has gained significant traction is the idea that females mate multiply to ensure that males with highly competitive sperm win the race to fertilize their eggs. For example, “good sperm” and “sexy sperm” (GS-SS) models (Curtsinger 1991; Yasui 1997) predict that traits involved in sperm competition should exhibit underlying heritable genetic variance, ensuring that polyandrous females secure indirect benefits from polyandry through the enhanced (sperm) competitiveness of their male offspring.

While evidence for heritability in sperm competitiveness is accumulating (Tregenza et al. 2009; Radwan 1998; Konior et al. 2005), we know much less about the genetic basis of FMM, despite this being the proposed mechanism underlying the selection of highly competitive sperm (reviewed by Evans and Simmons 2008). Specifically, according to GS-SS theory, multiple mating is the catalyst for “choice”, in the sense that highly polyandrous females have greater capacity for exercising postcopulatory selection than monandrous females. Consequently, and in line with quantitative genetic theory underlying traditional models of preference-trait coevolution (Kirkpatrick and Barton 1997), GS-SS theory makes explicit assumptions about the heritability of polyandry and corresponding (co)variances underlying male traits targeted by postcopulatory sexual selection (Evans and Simmons 2008).

**Figure 1:**
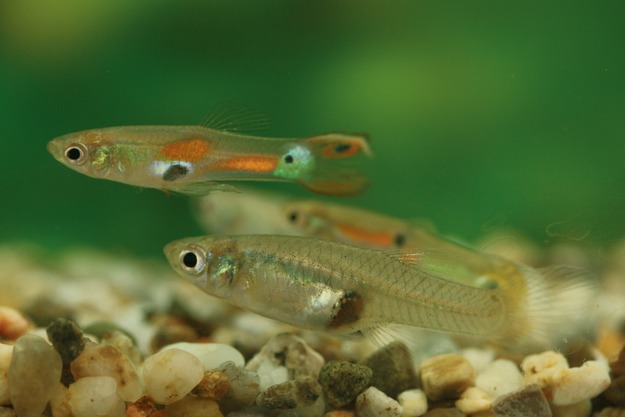
*Poecilia reticulata* male (top left) and female (bottom right).

In this article, we explore patterns of genetic variation in FMM in the guppy *Poecilia reticulata* (Fig. 1), a polyandrous livebearing fish exhibiting some of the highest recorded levels of FMM in any vertebrate (Hain and Neff 2007; Neff et al. 2008). As quantitative genetic studies of female reproductive traits are comparatively rare, particularly in vertebrates, we also explore patterns of genetic (co)variation in female fecundity and thereby explore possible covariances that may act as constraints to selection on FMM. Our article adds to just a handful of studies on insects (reviewed in Evans and Simmons 2008), birds (Reid et al. 2011), and mammals (McFarlane et al. 2011) that have tested for additive genetic variation in FMM. The consensus from many of these studies is that polyandry, or related measures of mating frequency, re-mating speed, and the propensity to engage in extra-pair matings, typically exhibits low levels of additive genetic variance and correspondingly low narrow-sense heritabilities. Nevertheless, the paucity of quantitative genetic studies on female mating behavior makes it difficult to draw broad conclusions on the potential for selection to act on FMM, thus motivating this study on guppies.

## Materials and Methods

### Breeding design

The fish used as the parental generation for the breeding design were approximately third-to-fourth generation descendants of wild-caught guppies from a feral population in Queensland, Australia. From this base population, we established a paternal half-sibling design by mating each of 40 sires to five dams (i.e. 200 dams in total). Matings were conducted using artificial insemination (Evans et al. 2003) to minimize possible differential maternal effects that can inflate sire variances (Kotiaho et al. 2003) and which are known to play a role in the guppy's mating system (Pilastro et al. 2004). Families were reared in full-sibling groups until the onset of sexual maturity (*ca*. 13 weeks), at which time females were removed from their natal tanks and reared in pairs in 2-L tanks until required. Importantly, only one female from each pair was used for the subsequent analyses to minimize common environmental effects. Rearing females in single sex groups ensured that they were virgin and therefore sexually receptive (Houde 1997) for the mating trials (below). The final dataset comprised *n* = 251 female offspring from 30 sires and 90 dams, although for some traits fewer observations were obtained. Final sample sizes are reported in Table 1.

**Table 1:** Number of offspring (*N*), trait means and standard errors (SE), number of sire (half-sib) and dam (full-sib) families (*n*) and variance components for sires (V_Sire_), dams (V_Dam_) and total phenotypic variation (V_p_). Narrow-sense heritabilities are presented separately for sires (*h*^*2*^_*sire*_) and dams (*h*^*2*^_*dam*_). Mean-standardized additive genetic variances (*I*_A_) were calculated as described in the main text. Note that significance values (*P*_sire_) come from mixed-effects models; FMM data were transformed (log*x*+1) to achieve a normal distribution prior to testing their significance (see main text). By contrast, estimates of *I*_A_ and *h*^2^ for FMM were calculated using untransformed data

Trait	*N*	Mean (SE)	*n* sires	*n* dams	V_Sire_	V_Dam_	V_p_	*h*^2^_sire_ (SE)	*h*^2^_dam_ (SE)	*I*_A_ (SE)	*P*_*sire*_
Standard length (mm)	251	24.09 (0.09)	30	90	0.25	∼ 0	1.99	0.50 (0.16)	∼ 0	0.002 (0.0007)	**0.001**
Fecundity (no. of eggs)	250	12.74 (0.30)	30	90	2.48	∼ 0	21.80	0.46 (0.20)	∼ 0	0.06 (0.026)	**0.003**
Female multiple mating (FMM)	250	3.8 (0.20)	30	90	0.27	0.34	10.07	0.11 (0.14)	0.13 (0.17)	0.07 (0.098)	0.40

### Estimating female mating frequency

Female offspring were used for behavioral trials at approximately 10 months of age (range in days: 191–455 days; mean ± SE = 313.90 days ± 4.35). In each trial, a focal virgin female was settled overnight in a mating arena (27 × 14 × 20 cm high, filled to 14 cm) containing a perforated plastic drinks bottle (1.5 L) housing four non-focal juvenile “dither” fish to promote normal swimming and social behavior in the test females (Barlow 1968). On the following morning, a single sexually mature male was selected at random from stock aquaria and placed in the mating arena with the female. If the female showed no sexual interest in the male (evident by a distinctive “glide” toward the displaying male; Houde 1997) within 5 min of him performing his first display, the male was replaced with a second male. If the female showed no sexual interest in this second male, the trial was terminated and a score of zero was assigned to the female. Where females successfully copulated with the initial male (evident when males performed a series of postcopulatory jerks signaling successful sperm transfer; Houde 1997), the male was immediately replaced with a subsequent male. This cycle was repeated until the female failed to exhibit a sexual response to two successive males (as described above), at which point the number of successful matings was recorded and the trial was aborted. This protocol was followed until all female offspring had been tested. Overall, 239 females successfully copulated with at least one male. At the end of each mating trial, females were killed and preserved in Dietrich's fixative (58% DI H_2_O, 30% ethanol, 10% formalin, 2% glacial acetic acid) until required for fecundity estimates (see below).

### Female fecundity

Preserved females were photographed and measured for standard length (distance from snout to the tip of the caudal peduncle). Dissections were performed on these specimens following previously described methods (Evans et al. 2011). Briefly, each female's body was positioned on a polystyrene base with the ventral side facing upward to reveal the ovary sac. Once exposed, eggs were carefully separated and counted to provide a measure of fecundity for each female.

### Quantitative genetic analyses

**Figure 2:**
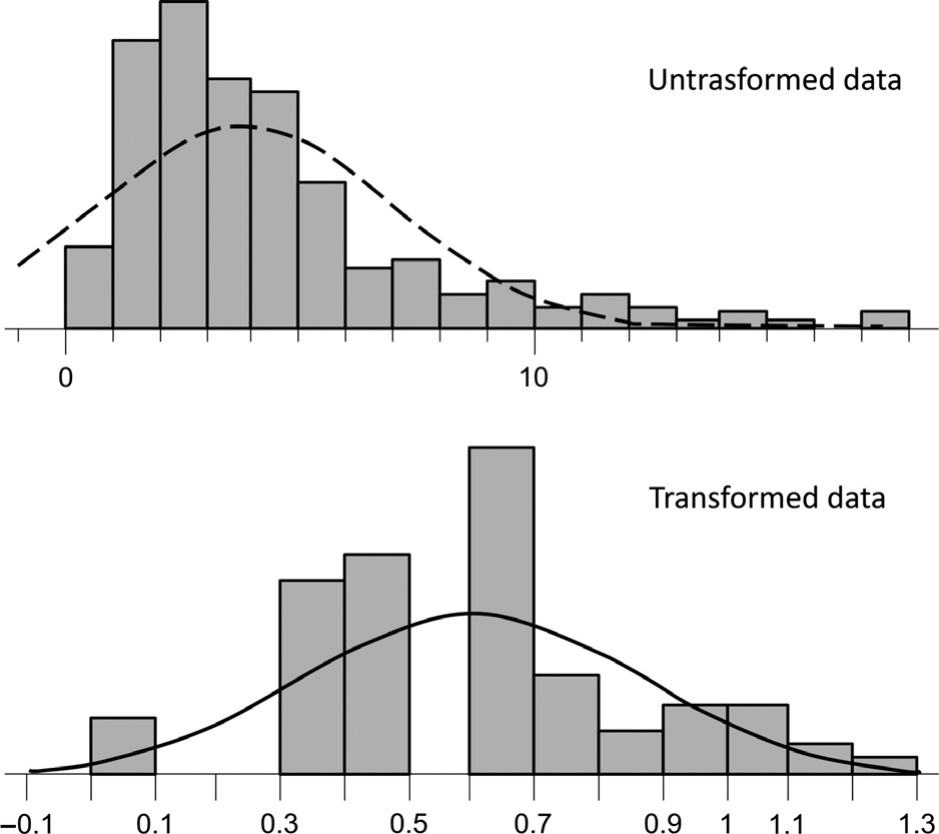
Frequency distribution of untransformed (a) and transformed (b) mating frequency data (FMM).

The heritability of individual traits and genetic correlations between them were estimated using restricted maximum likelihood procedures in the lme4 package of R 2.11.1 (R Development Core Team 2011). We fitted standard nested models for half-sib designs that included sire and dam identities as random effects. All data were tested for normal distributions prior to fitting the linear models. Data for FMM exhibited a moderately positive skewed distribution (skewness = 1.65) and were normalized with a log*x* + 1 transformation (skewness after transformation = −0.004; see Fig. 2 for raw and corrected distributions). Standard length and fecundity exhibited normal distributions. In the univariate tests, significance levels for the sire (additive) genetic variance components were determined using likelihood-ratio tests (Lynch and Walsh 1998). Narrow-sense heritabilities (*h*^2^) came from untransformed data (see Garcia-Gonzalez et al. 2012) and were estimated from the ratio of additive genetic to total phenotypic variance, while standard errors around these estimates were calculated by jackknifing across sire families (Roff 2006). Additive genetic covariances, required for the calculation of genetic correlations (*r*_G_) (Falconer and Mackay 1996), were estimated from subsequent bivariate models; standard errors around these estimates were calculated using the jackknife procedure (Roff and Preziosi 1994; Roff 2006). For some traits, data were missing (see above), thus generating a slight imbalance in the bivariate data required for genetic correlations. In these cases, we included only individuals for which both traits were available, so that variances and covariances were based on the same sample (see Lynch and Walsh 1998; p. 634). The significance of genetic correlations was estimated by comparing *z* scores for these estimates to the corresponding two-tailed significance levels from a standard normal probability table (Åkesson et al. 2008). Finally, estimates of evolvability come from mean-standardized additive genetic variances (*I*_A_), which estimate the expected percentage change in a trait under a unit strength of selection (see Hansen et al. 2011). Estimates of *I*_A_ were calculated using the formula *I*_A_ = V_A_/X^2^ (V_A_ = 4 × sire variance component; X = trait mean), while standard errors around these estimates were estimated by jackknifing across sire families.

### Protection of animal subjects in research

The work carried out in this study followed protocols and procedures that were reviewed and approved by the University of Western Australia's Animal Ethics Committee (approval number: RA/3/100/513).

## Results

Our analyses revealed considerable phenotypic variation in FMM, with females mating with up to 17 consecutive males during the mating trials (mean number of males mated ± SE = 3.8 ± 0.2; range = 0–17; Table 1). Despite the high variability, the proportion of total phenotypic variance in FMM attributable to additive genetic effects (*h*^2^) was relatively low and not statistically significant (Table 1). By contrast, both female fecundity and standard length exhibited significant additive genetic variances and correspondingly high narrow-sense heritabilities (Table 1). Female fecundity and standard length exhibited significant positive phenotypic covariance (*r* = 0.39, d.f. = 28, *P* = 0.034), but limited and non-significant additive genetic covariance (genetic correlation *r*_G_ = 0.21 ± 0.24; *z-*score and corresponding two-tailed significance level: *z* = 0.88, *P* = 0.38; see Table 2 for all genetic correlations).

**Table 2:** Genetic and phenotypic correlations for female reproductive traits in guppies. Genetic correlations and jackknife standard errors (in parentheses) are presented above the diagonal, while phenotypic correlations are presented below the diagonal for each pairwise relationship

Trait	Standard length	Fecundity	FMM
Standard length	–	0.21 (0.24)	0.66 (1.09)
Fecundity	0.39	–	0.18 (1.24)
Female multiple mating (FMM)	0.10	0.12	–

## Discussion

Our article adds to just a handful of studies documenting patterns of genetic variation in female mating frequency, which is important for evaluating GS-SS models that explain the evolution of polyandry. A central assumption underlying these models is that as multiple mating increases, so does the opportunity for postcopulatory sexual selection (for recent evidence see Collet et al. 2012), thus leading to the prediction of a genetic correlation between female mating frequency and the male traits targeted by postcopulatory sexual selection. Underlying this assumption is the expectation of non-zero heritability in FMM. While our estimate of additive genetic variance for FMM was not statistically significant, this is almost certainly attributable to sample size (we were constrained in the numbers of females tested within each full-sibling family, and the number of sire families that we could rear during the breeding phase). Nevertheless, our findings reflect an emerging pattern of low heritability in female mating frequency, and our estimate of *h*^2^ = 0.11 (± 0.14) for FMM is broadly comparable to narrow-sense (half-sib) heritability estimates for female mating frequencies and re-mating rates reported in insects (range 0–0.45, mean = 0.16; reviewed by Evans and Simmons 2008) and mammals (*h*^2^ = 0.06; McFarlane et al. 2011), as well as that for recurring matings between the same female and male pair (i.e. monandrous multiple mating) in beetles (*h*^2^ = 0.12; House et al. 2008).

For the purpose of assessing the evolutionary potential of FMM and comparing this measure with other published studies, we calculated *I*_A_ – the additive genetic variance scaled by the square of the trait's mean. Although as we noted above, the sample size of our experiment was necessarily modest given the relative intractability and space limitation associated with working with a relatively long-lived vertebrate, our estimate of *I*_A_ for FMM (0.07 ± 0.10) nevertheless offers a potentially informative effect size for FMM in guppies and thus gives some indication of the scope for selection on this trait. Our estimate of *I*_A_ is comparable to the median estimate of 0.1 for linear traits across 394 studies reported by Hansen et al. (2011), but an order of magnitude lower than their estimate of 0.95 for life-history traits. However, as noted by Hansen et al. (2011), an evolvability as low as the one we estimate for FMM may be hard to detect over a single generation, but can nevertheless generate large evolutionary responses over hundreds of generations.

Like the majority of studies testing for genetic variation in FMM (reviewed in Evans and Simmons 2008), we report high phenotypic variance, but limited additive genetic variance underlying this trait (but see Reid et al. 2011 for substantial additive genetic variance in the production of offspring sired by extra-pair males in song sparrows). Like many other studies of FMM, particularly on vertebrates, we were constrained to conduct a single point measure of mating frequency rather than a more comprehensive measure of lifetime (or long-term) mating frequency. Clearly, future studies that characterize more extensive measures of FMM may provide improved resolution of the genetic basis of this trait, although such a measure would be considerably challenging in vertebrate models with protracted reproductive lifespans such as ours. However, even with refined estimates of FMM, a second factor that may complicate our interpretation of quantitative genetic parameters in female mating frequency is that like many other traits involved in sexual selection, its expression is likely to depend on social context (reviewed by Moore and Moore 2006). Thus, as with other “interacting phenotypes” (Moore et al. 1997), the expression of the behavior (in this case willingness to mate repeatedly) may depend on context (e.g. male quality), which in turn determines the outcome of the interaction (i.e. FMM). The extent to which social context may have influenced our estimates of FMM is hard to judge, but given that female guppies exhibit heightened sexual responsiveness to males that are more sexually ornamented than previous partners (Pitcher et al. 2003), the decision to re-mate is likely to depend, at least to some extent, on an interaction between male and female genotypes. With the random selection of males in our experiment, our analysis took no account of such interactions. Future quantitative genetic studies of FMM may benefit from incorporating an interacting phenotype approach, in much the same way as proposed for studies of sexual conflict (Moore and Pizzari 2005) and social dominance (Moore et al. 2002).

Even with limited additive genetic variation in female mating rates, GS-SS processes may proceed via other mechanisms that facilitate postcopulatory female preferences (Evans and Simmons 2008). For example, in some insect taxa, the effect of sperm length on the outcome of sperm competition is dependent on the shape of the female's reproductive tract (García-González and Simmons 2007; Miller and Pitnick 2002; Pattarini et al. 2006), thus illustrating that the correlated evolution of FMM and male traits, explicit to current GS-SS models, may be unnecessary. Given the accumulating evidence for low levels of heritable genetic variation in FMM, our study further supports the call (Evans and Simmons 2008) for the broadening of GS-SS theory to account for alternative mechanisms of female postcopulatory preferences.
